# Use of Web 2.0 to Recruit Australian Gay Men to an Online HIV/AIDS Survey

**DOI:** 10.2196/jmir.1819

**Published:** 2012-11-06

**Authors:** Nathanaelle Thériault, Peng Bi, Janet E Hiller, Mahdi Nor

**Affiliations:** ^1^Direction régionale de santé publique de la Capitale-Nationale, Agence de la santé et des services sociaux de la Capitale-NationaleQuebec, QCCanada; ^2^Discipline of Public HealthAdelaide, South AustraliaAustralia; ^3^Faculty of Health Sciences, Australian Catholic University, Melbourne, VictoriaSchool of Population Health, University of AdelaideAdelaide, South AustraliaAustralia; ^4^The Hepatitis C Council of South AustraliaAdelaide, South AustraliaAustralia

**Keywords:** Internet, advertising, HIV, Australia, homosexuality, male

## Abstract

**Background:**

Continuous prevention efforts for human immunodeficiency virus (HIV) and acquired immune deficiency syndrome (AIDS) are recommended among those men who have sex with men (MSM). Creative use of e-technologies coupled with a better understanding of social networks could lead to improved health interventions among this risk population.

**Objective:**

The aims of the study were to (1) compare the impact of various advertising strategies on recruiting MSM participants to an online HIV/AIDS survey, and (2) explore the feasibility of using a social network service (SNS) for study advertising.

**Methods:**

A cross-sectional online survey was conducted in 2009. South Australian men over 18 years were invited to participate if they had had sexual intercourse with men in the previous year. A short questionnaire was used to collect demographics and information on sexual behavior, HIV history, use of the Internet for dating purposes, and sources of health information. The survey was promoted in community settings and online, including advertisements through social networks.

**Results:**

A total of 243 men completed the online survey during the 8-week data collection period. Online advertisements recruited 91.7% (220/240) of the sample. Conversely, traditional advertisements in the community recruited only 5.8% (14/240) of the sample. Ten volunteers were asked to advertise on their personal SNS application, but only 2 effectively did so. Only 18/240 (7.5%) of the respondents reported having learned of our study through the SNS application. In this sample, 19.3% (47/243) of participants had never been tested for HIV. Among the participants who had been tested, 12.8% (25/196) reported being HIV-positive. Regarding Internet use, 82.3% (200/243) of participants had dated online in the previous 6 months. Among the participants who had dated online, most (175/200, 87.5%) had found an Internet sexual partner and two-thirds (132/200, 66.0%) had had anal sex with these partner(s). Among men who had anal sex with an Internet partner, 68.2% (90/132) used a condom during sex.

**Conclusions:**

The MSM participants in this study had high-risk profiles for HIV and other sexually transmitted diseases (STDs), which highlights the need for ongoing health interventions among this group. In this study, the SNS marketing strategy did not appear to create a viral effect and it had a relatively poor yield.

## Introduction

South Australia (SA) is an Australian state with a population of 1,618,200 people of whom approximately 73% live in Adelaide, the capital city [1]. Information on the South Australian men who have sex with men (MSM) population is limited [2]. In 2001, it was estimated that 12,315 men in South Australia (2.1% of South Australian men over 16 years) were homosexual/bisexual [2].

In Australia, the MSM population constitutes the primary group affected by the human immunodeficiency virus (HIV) and acquired immune deficiency syndrome (AIDS) [2,3]. In South Australia, the MSM group accounts for 60%-70% of new HIV diagnoses [4,5].

Researchers have argued that new information technologies, such as the Internet, may have contributed to the global HIV epidemic [6]. The Internet appears to be particularly popular among MSM [7,8]. It is likely to have unique appeal to them because of the limited availability of venues where they can meet without fear of negative social consequences [9].

In an Australian online survey, most gay/bisexual male respondents had met someone in person after chatting online. The most frequent outcome of meeting following online contact was having casual sex (82%). However, many men reported having formed friendships (77%) and longer-term sexual relationships (41%) after such meetings [8]. In South Australia, an increasing proportion of MSM have reported using the Internet to look for male sexual partners [10]. In 2007, the Internet was the most popular venue for seeking partners, surpassing traditional venues such as bars and saunas [10].

The role of the Internet in the HIV epidemic among MSM is unclear [11,12]. A number of studies have shown an association between high-risk sexual behavior and using the Internet to look for sex among MSM [13-17]. The underlying causes are not completely understood [12,18], although there has been documented evidence of online promotion of high-risk sexual behaviors [6,19].

On the other hand, the Internet also offers great opportunities for expanding health promotion interventions [6,13,20]. In this unique setting, protective factors may operate simultaneously with risk factors [9]. For example, some websites targeting MSM already promote risk-reduction strategies, such as frequent HIV testing, limiting the number of sexual partners, and serosorting (the practice of choosing sexual partners with the same HIV status)[6]. In addition, it has been proposed that the Internet could provide better access to hard-to-reach groups of MSM who tend to receive less health promotion information, such as men who live in rural areas, men with culturally and linguistically diverse backgrounds, and men who do not overtly identify with the gay community [7,18,21].

Social network services (SNSs), such as Facebook, MySpace, Orkut, LinkedIn, Hi5, and BeBo,are increasingly popular. Using SNSs to recruit for health studies has emerged to be a cost-effective tool in recent years [11,22].

Facebook was the focus of this study. At the time of this study, Facebook had more than 300 million users worldwide including 7 million Australian users (6.2 million over 18 years), and its popularity was growing [23-25].Within 50 miles of the Adelaide city center, there were more than 500,000 active Facebook users (364,000 over 18 years) including 157,000 adult men at the time of study [25].

Public health approaches to health promotion can benefit from taking advantage of the possibilities created by the Internet, especially by SNSs [23].A better understanding of Internet use and social networks among MSM could lead to improved health promotion initiatives in this specific population.

This study aimed to (1) compare the impact of various advertising strategies on recruiting MSM participants to an online survey, and (2) explore the feasibility of using an SNS for study advertising.

## Methods

A cross-sectional online survey was performed between June 26 and August 21, 2009. It was advertised as the “SA Men Online Survey” in both community and online settings.

### Study Advertising

#### Advertising in the South Australian Gay Community

The survey, endorsed by the AIDS Council of South Australia (ACSA) and approved by the University of Adelaide Human Research Ethics Committee, was advertised in a newspaper for the South Australian gay community (*Blaze*) and through posters and business-sized cards distributed in the Adelaide area in gay venues and gay health service clinics (Table 1). The materials contained the study webpage URL that potential respondents were encouraged to access.

**Table 1:** Media utilized for study advertising.

Media		Description	Location
**Online advertising**			
	Web banner	Continuous banner placement	acsa.org.au; Gaydar.com.au
	Invitation in chat room	Message displayed on a few occasions	Gaydar.com.au
	Email signature	Banner attached to emails of 3 staff members	Email to potential respondents
	E-newspaper	Weekly e-news sent via email to 800 male members	Email to potential respondents
	SNS	Via webpage, ad placement, and advertisement on social networks	SNS application
**Community advertising**			
	Gay newspaper (*Blaze*)	8500 copies of a single issue, color ad on 1/8 of A4 page	Adelaide city center and two other cities
	Business-size cards	250 color cards	Sex-on-premises venues (2), bar (1), general practice clinic (1), HIV/AIDS support service (1)
	Posters	50 color posters, A4-size	As above
	Word of mouth	Word of mouth encouraged on materials	Community

#### Online Advertising

**Figure 1:**
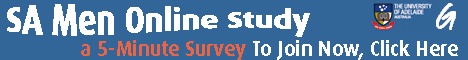
Banner advertisement used to recruit potential men who have sex with men for an online HIV/AIDS survey, South Australia, 2009.

Gaydar.com.au is a UK company with a repertoire of chat rooms dedicated to MSM based on geographic location. A Web banner was placed on the home pages for “South Australia-Adelaide” and “South Australia-Rest.” The Web banner contained the University of Adelaide and the ACSA/Gay Men’s Health (GMH) logos and read, ‘‘SA Men Online Study. A 5-Minute Survey. To Join Now, Click Here”(Figure 1).

Educators from the ACSA/GMH offer peer-based information and support inthe Gaydar.com.au chat rooms as part of their routine duties. They provided information about the study and were instructed to refer the chatters to the study coordinator to address concerns or complex questions. During the study period, educators displayed a general message containing study name, study URL, and an invitation to participate in the South Australia-Adelaide chat room of Gaydar.com.au.

In addition, the study banner was displayed on the ACSA/GMH website. Approximately 800 male members subscribe to an e-news service provided by the ACSA/GMH and they were also invited to participate in the study. In addition, three educators from the ACSA/GMH attached the study banner to their email signature block during the study period.

#### SNS Advertising

Ten MSM were approached initially to advertise the study using their personal Facebook account and three agreed to participate. They met with the project coordinator and were provided with written information on study advertising using their SNS. When the survey was launched, they were contacted by the project coordinator via email to provide information about posting the study name and URL link on their personal page. No incentive was provided, but they were offered the results at the conclusion of the study.

Ideally, volunteers would have been identified on the basis of objective social leader characteristics using appropriate methods [26]. However, our request to advertise on their personal SNS may have been perceived as intrusive. As a result, recruitment was made on a voluntary basis only.

A webpage entitled “SA Men Online” was also placed on Facebook free of charge with page access restricted to people over 18 years. As a result, people could not access this page directly from an external website or from a search engine. Only logged-in users of that particular SNS over 18 years (as stated in their profile) could potentially view it.

Volunteers were asked to identify themselves as fans of this Facebook page. Updates on study progression, number of respondents, and some general comments from previous respondents were posted on this webpage.

To reach additional respondents, a banner advertisement was also placed on the Facebook sidebar for a 2-week period targeting men over 18 years, living within an 80-kilometer radius of Adelaide city center, who were interested in men. An estimated 1540 users were targeted by the advertisement. Clearly, this strategy would only access individuals who were willing to identify their sexual orientation on a SNS.

### Online Survey

#### Design

The target population consisted of all South Australian men over 18 years who have had sex (oral/anal) with men in the previous year. A convenience sample was drawn from this population and a cross-sectional survey was conducted over an 8-week study period in 2009.

#### Institutional Review Board Approval and Informed Consent Process

The study was approved by the Human Research Ethics Committee of the University of Adelaide, Australia.

Before accessing the survey questionnaire, all potential respondents had to access the official study webpage placed on the University of Adelaide website. The informed consent form appeared on the main webpage and contained details about survey length, data storage, and study investigators. It explained possible positive and negative outcomes related to study participation. At the end of this page, men who agreed to participate could signify so by clicking a button that lead to the questionnaire screen. The online questionnaire did not collect identifiable personal information such as name, date of birth, or email address.

Electronic survey data were stored in a locked office, in an appropriate password-protected computer on a password-protected network within the firewalled computing environment of the Discipline of Public Health, University of Adelaide, Australia.

#### Development and Pretesting

The questionnaire was built using validated questions from previous surveys [8,10,27]plus additional questions. A paper version of the questionnaire was pretested with 5 MSM community volunteers. Minor changes were made to the questionnaire in accordance with their feedback.

The final questionnaire collected demographic data (age, educational level, and postal code), sexual identity, use of websites for meeting partners (types of websites, number of partners met, and condom use with last partner), HIV history (status and date of last test), usual sources of health information, and information on study advertising media. The questionnaire included additional items on each of the selection criteria (gender, place of residence, and sex with men history) and a comments section.

The commercial software chosen for this study significantly facilitated the online survey design process because it did not require technical expertise in computer programming. The company also collected respondents’ data via secure sockets layer (SSL) encryption and they initially hosted the database. The company ensured that only the research team would be able to access the data via password. Time constraints and associated costs were considered in choosing the software package.

The questionnaire was tested using both Internet Explorer and Firefox Web browsers. At a pretesting stage, an email containing the survey URL was sent to 5 designated respondents who took part in the survey and provided comments.

#### Recruitment Process

The survey was open to all visitors to the study webpage, which was not password-protected. The selection criteria for study participation were highlighted clearly.

The survey was advertised offline and online. Regardless of the adverting strategy, ultimately all respondents were directed to the study webpage to complete the questionnaire online.

#### Survey Administration

The survey was made accessible exclusively through the webpage created on the University of Adelaide website to facilitate the consent process. The study webpage was placed under “Research Projects” in the Discipline of Public Health. Because of the number of steps (9 successive clicks) required to access the study webpage from the main university webpage, it appeared relatively unlikely that people would access it without having been exposed to any study advertisements.

The survey itself was voluntary. Participants were informed that results would be posted online and, apart from links provided to useful resources on the website, no other incentive was offered.

The survey was available online for an 8-week period. The questions were displayed in identical order to all respondents. Depending on previous responses in the questionnaire, some questions were skipped. The questionnaire was kept short to encourage maximal participation.

The questionnaire comprised 8 screens so that questions that could potentially be skipped were placed on separate pages. There were 1-5 items per screen and 17 items in total. No item was mandatory and incomplete questionnaires could be submitted. Participants were able to change their answers from previous pages using a “previous” button.

#### Response Rates

During the study period, there were 544 unique visitors to the study webpage on the University of Adelaide website. However, the software company used to launch the survey did not provide figures for unique visitors to the questionnaire itself. Specifically, the company did not record the number of respondents who exited without completing or submitting the first page of the questionnaire. Therefore, the participation rate could not be calculated.

The completion ratio (ratio of the number of people who submitted the last survey page divided by the number of people who submitted the first survey page) was 0.946 (247/261).

#### Considerations Regarding Potential Multiple Entries from the Same Individuals

Given the topics assessed in the survey, for ethical reasons the research team was unaware of the Internet protocol (IP) addresses of the respondents’ computers and the team did not use cookies. However, the company that provided the software did use cookies and was able to identify the IP addresses. The company noted that this information was necessary for the proper functioning of the online software and that it was used in an aggregated manner for administrative purposes only.

Multiple entries from a single IP address were not restricted because more than one person in a given household may have been eligible and attempted to participate. Secondly, one IP address could be shared by several computers. Moreover, restricting multiple submissions from the same IP address did not necessarily prevent individuals from submitting multiple surveys from computers with different IP addresses.

However, the database was screened for potential duplicate submissions. Questionnaires with an identical combination of age, educational level, and postal code were checked for the dates of last HIV test and HIV status. None were identical; therefore, all files were retained in the final analysis.

An examination of the time for completion did not reveal any aberrant results. The shortest completion time was 1.17 minutes, but the answers were credible. Therefore, no survey was eliminated because of a too-short completion time. Median time for survey completion was 3.27 minutes.

Surveys were checked for completion. Seven respondents did not complete the last page, but they did complete the 7 previous pages. Overall, given that some questions were not applicable to all respondents, all surveys had at least 75% completion of applicable questions and all were kept in the analysis.

### Statistical Analysis

Descriptive statistics were performed using Predictive Analytics Software (PASW) Statistics 17.0 software [28]. No method (ie, weighting of items or propensity scores) was used to adjust for the non-representative sample.

## Results

In total, 261 questionnaires were completed during the 8-week data collection period (June 26 to August 21, 2009). Eighteen were excluded because they did not meet the inclusion criteria based on gender (7/261, 2.7%), age (1/261, 0.4%), place of residence (1/261, 0.4%), or history of sex with men in the previous year (9/261, 3.4%). The remaining 243 surveys formed the database for the final analyses.

### Study Advertising

**Figure 2:**
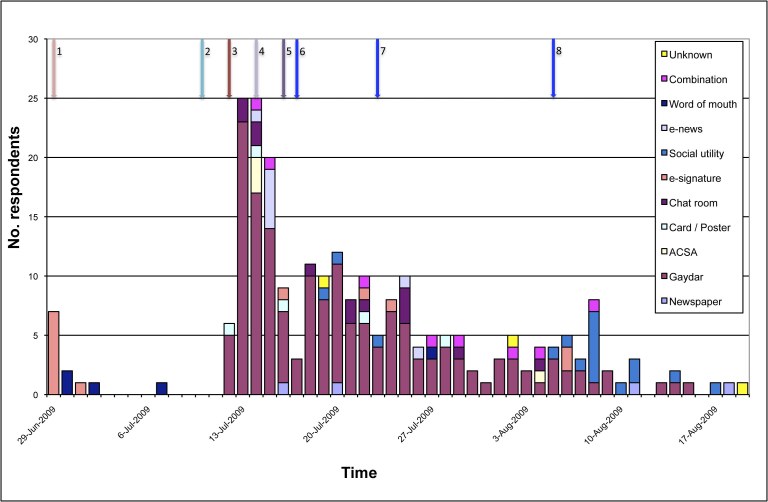
Number of men who have sex with men recruited to an online HIV/AIDS survey by type of media between June 26 and August 21, 2009, in South Australia.

Over the study period, advertisements were introduced in a staggered manner so it was possible to examine the relative success of each recruitment strategy (Figure 2). The advertising was limited in the first 2 weeks to allow for eventual technical adjustments. Overall, recruitment was low in the final 10 days and data collection was stopped.

In Figure 2, the arrows refer to the dates that the advertising strategies were implemented. They are:

1. Web banner attached to e-signature of 3 staff members of the ACSA/GMH.

2. Card and poster distribution in gay venues and health clinics in the Adelaide area.

3. Web banner placement on Gaydar.com.au and message display in chat room of Gaydar.com.au.

4. E-news sent weekly by ACSA/GMH to 800 members and Web banner placement on the ACSA/GMH website.

5. Advertisement in newspaper for gay men.

6. SNS: Study name and URL posted on personal Facebook page by volunteer #1.

7. SNS: Study name and URL posted on personal Facebook page by volunteer #2.

8. SNS: Ad placement on Facebook.

When men were asked about the media that alerted them to the study, 232 out of the 240 men (96.7%) reported only 1 source and 8 men reported 2 sources. The advertisement banner posted on Gaydar.com.au attracted 70.0% (168/240) of respondents with an additional 6.3% (15/240) recruited through the chat room on Gaydar.com.au. Eighteen (7.5%) respondents reported having learned of our study through the SNS application. The ad launch on that application recruited some respondents in the first week, but plateaued in the second week.

Overall, the online advertising made in chat rooms, on the SNS application, via banners and emails recruited 220/240 (91.7%) respondents. Community advertising through posters, business cards, the newspaper, and word of mouth, recruited only 14/240 (5.8%) respondents. In all, 6/240 (2.5%) reported having been exposed to both online and offline media.

Data (eg, impressions and click-throughs) regarding the banner advertisements could not be obtained because specific arrangements were not made with ACSA/GMH and Gaydar.com.au to track such information.

### SNS Advertising

During the study period, 2 of the 3 volunteers who had initially agreed to advertise the study on their SNS advertised the study effectively. They had a cumulative social network of approximately 100 friends, which is relatively small considering the average Facebook user has 130 friends [29]. In addition, the webpage created on the SNS application did not have high access rates.

Over 14 days, the advertisement on the SNS was displayed 55,000 times with 53.5% of total impressions being directed toward the 18-24 year group, and an additional 22.0% toward the 25-34 year group. Daily, 400-870 distinct users were shown the advertisement. On a given day, individuals who were shown the advertisement would see it 5 times on average. Only 31 people clicked on the advertisement itself, thus 0.06% (31/55,000) of all impressions resulted in a click. This approach incurred relatively modest fees of Aus $12.36 for 31 clicks (average Aus $0.40 per click).

### Online Survey

#### General Characteristics of the Sample

Table 2 describes characteristics of survey respondents. The age range was between 18 and 68 years, with a median age of 34 years. Most respondents (140/243, 57.6%) were aged 25-44 years. The sample was highly educated, with 119/242 (49.2%) having completed a university degree or a College of Advanced Education (CAE) course.

**Table 2:** Characteristics of South Australian MSM recruited online, 2009.

Characteristics^a^		n	%
**Age (n = 243)**			
	18-24	50	20.6
	25-34	73	30.0
	35-44	67	27.6
	45-64	52	21.4
	≥ 65	1	0.4
**Education (n = 242)**			
	Less than or up to 3 years of high school/Year 10	22	9.1
	Year 12/South Australian Certificate of Education (SACE)	52	21.5
	Tertiary diploma or trade certificate/Technical and Further Education (TAFE)	49	20.2
	University/College of Advanced Education (CAE)	119	49.2
**Area of residence (n = 241)**			
	Metropolitan Adelaide	212	88.0
	Rural area	21	8.7
	Remote area	8	3.3
**Socioeconomic Index for Area (SEIFA)** ^**b**^ **(n = 241)**			
	1st quintile (most disadvantaged)	57	23.7
	2nd quintile	41	17.0
	3rd quintile	59	24.5
	4th quintile	49	20.3
	5th quintile (least disadvantaged)	35	14.5
**Sexual identity (n = 242)**			
	Gay/homosexual	196	81.0
	Bisexual	43	17.8
	Heterosexual	0	0
	Unknown/other	3	1.2
**Lifetime HIV test history (n** **= 243)**			
	Never tested	47	19.3
	Tested at some point	196	80.7
**Declared HIV status according to last test result (n = 196)**			
	Negative	170	86.7
	Positive	25	12.8
	Unknown	1	0.5
**Declared time of last HIV test for tested men** **without a HIV-positive result (n = 171)**			
	< 3 Months	43	25.2
	3-6 Months	39	22.8
	7-12 Months	34	19.9
	1-2 years	24	14.0
	> 2 years	31	18.1
**Sources of health information (** **n = 234)** ^**c**^			
	Books	47	20.1
	Family and friends	49	20.9
	Gay media	60	25.6
	Gay Men’s Health website	63	26.9
	Gay Men’s Health (resources other than the website)	33	14.1
	Health care provider	97	41.5
	Health department	36	15.4
	Internet	160	68.4
	Men’s magazine	71	30.3
	Newspaper/news magazine	53	22.6
	Professional education	54	23.1
	Radio	25	10.7
	Television	81	34.6

^a^The number of respondents answering each question varied slightly because of missing values; question related to HIV status was automatically skipped for 47 untested men.

^b^The Index of Relative Socioeconomic Disadvantage (IRSD) includes attributes such as low income, low educational attainment, high unemployment and jobs in relatively unskilled occupations, and refers to the area in which a person lives [30].

^c^More than one option possible.

Most participants (212/241, 88.0%) resided in metropolitan Adelaide, but men from rural (21/241, 8.7%) and remote (8/241, 3.3%) South Australia were also represented. A socioeconomic index was obtained from the area of residence of participants. There was no clear gradient, but proportionally fewer men (35/241, 14.5%) were in the least disadvantaged quintile than the most disadvantaged quintile.

Most respondents (196/242, 81.0%) considered themselves as homosexual/gay whereas 43/242 (17.8%) described themselves as bisexual.

Regarding HIV testing, 47/243 (19.3%) men reported never having been tested. Among men who had been tested at some time, 25/196 (12.8%) reported being HIV-positive, 170/196 (86.7%) reported being HIV-negative, and 1/196 (0.5%) reported an unknown status according to the last test result. Among tested men who did not report an HIV-positive result, 43/171 (25.2%), 39/171 (22.8%), and 34/171 (19.9%) participants reported having had an HIV test in the previous 3 months, previous 3-6 months, and previous 7-12 months, respectively. Fifty-five participants (32.1%) had been tested more than a year previously.

Participantsreported various sources of health information, with the Internet as the most noted source (160/234, 68.4%). A health care provider (97/234, 41.5%), television (81/234, 34.6%), men’s magazines (71/234, 30.3%), and Gay Men’s Health website (63/234, 26.9%) were also frequently reported.

#### Online Dating and Sex

Regardingonline dating, 200/243 (82.3%) participants reported having looked for male sexual partners on the Internet in the previous 6 months (Table 3). These men were further asked about their online dating habits and related sexual behavior. Most (175/200, 87.5%) had met at least one male sexual partner through the Internet in the previous 6 months. Approximately half of the participants who had met Internet partners (86/175, 49.1%) had found 2-5 partners. Of the participants who reported having looked for male partners online, 132/200 (66.0%) had anal sex with an Internet partner, 68.2% (90/132) of whom reported having used a condom during their most recent sexual encounter. In the subgroup of participants who reported unprotected anal sex during their last intercourse, 9/42 men (21%) had never been HIV tested. Among tested participants, 5/33 (15%) reported being HIV-positive. The only 2 participants surveyed who reported having had more than 50 Internet partners also reported having unprotected anal sex during their last sexual encounter.

**Table 3:** Online dating and sex among South Australian MSM, 2009.

Characteristics^a^		n	%
**Online dating with males in previous 6 months (n = 243)**			
	Yes	200	82.3
	No	43	17.7
Websites utilized for dating (n = 200)^b^			
	Gaydar.com.au	178	89.0
	Manhunt	109	54.5
	Squirt	50	25.0
	Facebook	16	8.0
**Number of male sexual partners met through Internet in previous 6 months (n = 200)**			
	None	25	12.5
	1	39	19.5
	2-5	86	43.0
	6-10	29	14.5
	11-50	19	9.5
	> 50	2	1.0
**Anal sex with a male partner met through the Internet in previous 6 months (n = 200)**			
	Yes	132	66.0
	No	68	34.0
**Condom use during last anal sex with a male partner met through the Internet (n = 132)**			
	Yes	90	68.2
	No	42	31.8

^a^The number of respondents answering each question varied only because of automatically skipped questions.

^b^More than one option possible.

## Discussion

### Principal Results

Online advertising was a less costly and more efficient approach to recruit in comparison to community advertising during the relatively short recruitment phase. Our findings suggest that in this gay community that is well educated and mostly urban, online advertising is likely to be a more successful method of recruiting participants to an online survey unless the community approach is considerably intensified.

Our results also indicate that MSM who participated in this study constitute a high-risk group for sexually transmitted diseases (STDs) and HIV infections. For instance, nearly half (102/218, 46.8%) of the non-HIV-positive participants had not had an HIV test in the previous year. In addition, 13% of the participants who underwent an HIV test were HIV-positive. This proportion is higher than that of previous surveys [8,10] and is suspected to be higher than the South Australian MSM population HIV prevalence rate overall [2]. Thus, men recruited on dating websites may be at higher risk for HIV infection [21]. Furthermore, participants were recruited with the active involvement of an HIV/AIDS organization that may have reached a higher proportion of HIV-positive respondents. Finally, one-third of participants had unprotected anal sex during last intercourse with their Internet partner.

In this online recruited sample, the Internet was the most popular source of health information, which highlights the need for continuing and developing creative Internet-based interventions.

### Comparison with Prior Work

Regarding study advertising, previous online surveys assessing HIV risk have mainly recruited MSM from sites that were primarily gay-identified or that were sexual meeting sites [18,31-35]. As mentioned previously, statistics regarding banner placement on Gaydar.com.au and the ACSA/GMH website could not be obtained for comparison.

In an American study, MSM were recruited to an online survey through SNS advertisements. The click-through rate for the banner advertisements on MySpace.com was0.37% [11]. The researchers concluded that this strategy allowed recruiting large numbers of MSM (n = 9005) in a short period of time of approximately 1 month [11]. These figures differ from ours; our click-through rate for the banner advertisements on Facebook was 0.06% and the overall SNS marketing strategy had a relatively poor yield with only 18 participants recruited in 8 weeks.

Comparison with previous Australian studies can be made in regards with our survey findings. The Gay Community Periodic Survey (GCPS), a pen-and-paper survey in Australia, was conducted among MSM of Adelaide in 2007. Findings from that study differed to ours, mainly in regards to lifetime uptake of HIV tests (in the GCPS, 10.4% of men had never been tested for HIV versus 19.3% in this study) and the reported HIV status (5.7% versus 10.3% of men reported an HIV-positive status). These differences could be due to chance, given our limited sample size. However, online and offline recruited samples are known to have different characteristics [36]. Therefore, it cannot be excluded that a higher-risk group was selected in our online sample. In addition, the anonymity provided by the Internet may have encouraged more men to divulge their positive HIV status because the Internet may be more favorable for respondents to disclose information on sensitive topics [37].

In Private Lives*,* an online survey conducted across Australia in 2005 [8], the proportion of men who had been tested for HIV at least once (78%) was similar to our findings (80.7%). Among tested men, nearly two-thirds had been tested in the previous year in both studies (65% in Private Lives versus 67.8% in our study) [8]. Similarities in the advertising and survey methods of both online surveys could explain these figures. However, in Private Lives, a lower proportion of HIV-tested men had a positive test result (9.7%) in comparison with 12.8% from our findings [8].

### Strengths

This is one of the few studies [11] to make use of SNS as a practical tool to recruit MSM in a health research project, which makes it innovative. It shows that a paid SNS advertisement is easy to implement, but that the utilization of naturally existing social networks could be a challenge. The creation of a public page on a SNS application is also an interesting strategy, although stimulating interaction around it warrants detailed planning.

Interestingly, a search on October 27, 2009, for keywords in the medical subject headings (MeSH) list did not return accurate results (using “social networking,” “social network,” “network,” “online network,” “network service,” “social network service,” “Facebook,” and “MySpace”). The closest result was related to the idea of “social support.” This reinforces the idea that the concept of SNS is relatively new in the health research field and needs to be further considered given its impact and omnipresence in everyday life.

This pilot project was designed to make an efficient use of time and material resources. Responses were obtained from 243 respondents within an 8-week time frame. The total additional expenditure incurred by the research team, excluding human resource expenditure and costs related to regular ongoing activities at the ACSA, was approximately Aus $460 which is less than Aus $2 per respondent. One-third of the total amount was paid to the software company over a 3-month period. Most of the expenditure related to printing of materials that were distributed in the community. In this study, online advertising was relatively inexpensive because the ACSA already had an agreement with Gaydar.com.au and study advertising on that website did not incur additional costs. Fees incurred with advertising on the SNS were minimal given the relatively poor yield of that strategy.

### Limitations

Limitations of this study should be acknowledged. The online self-selected sample, mostly recruited on a MSM dating website, may not be representative of the whole MSM population of South Australia [38,39]. However, it does capture a high-risk group of men that could be reached via online interventions as part of a comprehensive health promotion strategy.

The deliberately short questionnaire could not fully assess all factors of interest. For instance, in regards to sexual behavior, men were not asked about the presumed status of their partner nor were they asked about the use of risk-reduction strategies. It is possible, among men who did not use a condom at last anal sex with an online partner, that their decision was based on assumptions of concordant HIV status [40].

For ethical concerns related to the survey-specific content (ie, HIV status) the research team deliberately chose not to gather the respondents’ IP addresses and to not use cookies. Therefore, potential multiple entries coming from a single computer were not restricted. The database was screened for potential duplicate submissions based on an identical combination of answers to some key questions. However, this approach does not prevent intentional deception. This may have biased the study results.

Some technical limitations were encountered during this project. Firstly, respondents had to report the media that made them aware of our survey. Ideally, it would have been preferable to be able to directly track the pathway of each respondent.

Secondly, there were limitations associated with the creation of the study webpage on a university website. On the one hand, having the webpage on a university website may have reassured some respondents of the project legitimacy. However, this resulted in a lack of flexibility regarding the webpage features, such as its location (and correspondingly long URL) and the automatic display of images in the sidebars of the webpage.

Finally, the use of a commercial software, although user friendly and reasonably priced, imposed some constraints, such as not providing all the survey traffic statistics that would have been of interest (eg, the number of people who dropped out without completing the first questionnaire page).

Because this study did not appear to create a viral effect, this warrants reflection on the conditions required to make study participation more attractive to potential respondents and their networks [23]. The addition of a pleasant interactive Web tool could facilitate this phenomenon. This could have various formats: an interactive story, a “test-your-knowledge” tool, a decision-support tool [41], a game related to the topic, among others. Public health researchers have been successful in creating a viral marketing strategy [41,42].

### Conclusion

The MSM who were surveyed in this online study constitute a high-risk group in regards to STDs and HIV infections. Our findings highlight the need for continuous interventions among this group.

This study explored some avenues for health care research in the MSM population using SNSs. In this study, the SNS marketing strategy did not appear to create a viral effect and it had a relatively poor yield. Nevertheless, it illustrates that SNSs can be practically used for public health research.

## Abbreviations

ACSA: AIDS Council of South Australia
AIDS: acquired immune deficiency syndrome
CAE: College of Advanced Education
GCPS: Gay Community Periodic Survey
GMH: Gay Men’s Health
HIV: human immunodeficiency virus
IP: Internet protocol
IRSD: Index of Relative Socioeconomic Disadvantage
MeSH: medical subject headings
MSM: men who have sex with men
PASW: Predictive Analytics Software
SA: South Australia
SACE: South Australian Certificate of Education
SEIFA: Socioeconomic Index for Area
SNS: social network service
SSL: secure sockets layer
STD: sexually transmitted disease
TAFE: Technical and Further Education
